# The Role of Soil Microbial Consortia in Sustainable Cereal Crop Residue Management

**DOI:** 10.3390/plants13060766

**Published:** 2024-03-08

**Authors:** Arman Shamshitov, Gražina Kadžienė, Skaidrė Supronienė

**Affiliations:** 1 Laboratory of Microbiology, Institute of Agriculture, Lithuanian Research Centre for Agriculture and Forestry, Instituto al. 1, Akademija, LT-58344 Kedainiai, Lithuania; skaidre.suproniene@lammc.lt; 2 Department of Soil and Crop Management, Institute of Agriculture, Lithuanian Research Centre for Agriculture and Forestry, Instituto al. 1, Akademija, LT-58344 Kedainiai, Lithuania

**Keywords:** cereal crop residue, decomposition, microbial community, agricultural practices, lignocellulolytic microorganisms

## Abstract

The global escalation in cereal production, essential to meet growing population demands, simultaneously augments the generation of cereal crop residues, estimated annually at approximately 3107 × 10^6^ Mg/year. Among different crop residue management approaches, returning them to the soil can be essential for various ecological benefits, including nutrient recycling and soil carbon sequestration. However, the recalcitrant characteristics of cereal crop residues pose significant challenges in their management, particularly in the decomposition rate. Therefore, in this review, we aim to summarize the influence of different agricultural practices on enhancing soil microbial decomposer communities, thereby effectively managing cereal crop residues. Moreover, this manuscript provides indirect estimates of cereal crop residue production in Northern Europe and Lithuania, and highlights the diverse roles of lignocellulolytic microorganisms in the decomposition process, with a particular focus on enzymatic activities. This review bridges the knowledge gap and indicates future research directions concerning the influence of agricultural practices on cereal crop residue-associated microbial consortia.

## 1. Introduction

The definition of crop residue has transformed in the last few decades, reflecting shifts in agricultural practices, technological progress, and a deeper understanding of its role in promoting sustainable agriculture and the environment. Earlier, the main focus of the definition was centered on the vegetative remnants left in fields after harvesting the main crop. It was frequently considered as waste and required removal from the field in order to facilitate preparation for the following planting season, particularly since it was viewed as an impediment to conventional tillage practices [1]. In contrast, modern agriculture redefines crop residue as a plant material deliberately retained in the fields, aligned with conservation agriculture practices. Furthermore, redefinition has expanded to reflect its potential impacts on carbon sequestration, and climate change mitigation, and is an important resource for the generation of bioenergy and the establishment of a circular economy [2,3,4]. Northern Europe, including Nordic countries, the UK, Ireland, and Baltic states plays a significant role in cereal grain production encompassing wheat, barley, maize, oats, and rye within the European Union [5,6].

It is well-known that incorporating or retaining cereal crop residue on the soil surface has numerous advantages in improving soil quality. Hence, the large-scale removal of crop residues from fields might have a detrimental impact on soil quality and productivity [7,8] by lowering total nitrogen (N) levels and soil organic carbon (C) [9,10], enhancing wind and water erosion [11], and decreasing soil microbial activity [12]. Furthermore, it is important to acknowledge the influence of C, N, cellulose, lignin, and polyphenols on the rate at which nutrients are released from agricultural residues. These decomposition products have a significant impact on the absorption rate of nutrients by crops [13]. The different approaches of cereal crop residue utilization include livestock fodder, compost, biogas, biochar or biofuel production and incorporation into the field. Among these approaches, incorporation is considered to be a better option since it manages a large quantity of residue while improving soil health [14,15].

The soil microbial community plays a crucial role in crop residue decomposition, carbon processes, and nutrient cycle in the soil. To break down crop residue components effectively, a collaborative effort of various hydrolytic and oxidative enzyme families is essential. These enzymes are produced by lignocellulose-degrading microorganisms and work together in a coordinated manner to carry out multiple oxidative, hydrolytic, and non-hydrolytic activities. In essence, they function as a synergistic cocktail with complementary actions to achieve the breakdown process [16,17,18].

The influence of residue management and tillage treatment on crop production, and soil fertility, is known to be highly dependent on the pedoclimatic conditions (soil type, temperature, precipitation, sunlight, wind, and humidity). Therefore, this manuscript provides indirect estimates of cereal crop residue production in Northern Europe and Lithuania, assessing quantities at both regional and state scales. We hypothesize that conservation practices can enhance the development of a stable microbial community for decomposing crop residues. This comprehensive review aims to address the knowledge gap in understanding how conventional and conservation agricultural practices influence soil microbial communities involved in cereal crop residue decomposition. Moreover, this review captures soil microorganisms encompassing lignocellulose decomposition and their associated enzymatic activities, both in individual and consortium settings, which remains lacking.

## 2. Estimates of Cereal Crop Residue Production in Lithuania and Northern Europe

Crop residue is typically recognized as one of the major and most significant sources of organic matter in soil. According to Turmel [7], major cereal crops including maize (*Zea mays* L.), wheat (*Triticum aestivum* L.), barley (*Hordeum vulgare* L.), sorghum (*Sorghum bicolor* L.), and rice (*Oryza sativa* L.) produce considerable amounts of crop residue. In 2021, the total worldwide area harvested for these crops was approximately 221 million hectares for wheat, 206 million ha for maize, 165 million ha for rice, 49 million ha for barley, and 41 million ha for sorghum [19]. Alternatively, it is estimated that the annual worldwide production of postharvest residues from cereal crops in 2050 might reach 2.004–2.116 Gt [20]. Differences in cultivated area, crop types, and yields vary significantly among countries worldwide, primarily driven by factors such as climate conditions, specific soil characteristics, and diverse farming practices. Among all crops, cereals stand out as the most crucial ones in terms of both cultivated area and production.

Crop residue production has increased substantially all over the world in recent years. Hence, according to the estimates, the total amount of cereal crop residue production has increased by 16.90% in Northern Europe since 2001 (Table 1). The data in Table 2 show that in 2021, the annual residue production of 6373.3 Kt (kiloton) of wheat, 750.6 Kt of barley, 170.3 Kt of oats, 104.7 Kt of maize, and 95.2 Kt of rye in Lithuania. It is worth noticing that wheat residue accounts for about 85% of the main cereal crops’ residue. The total production of cereal crop residue in Lithuania is estimated at 7.5 Mt (megaton), whereas in Northern Europe, it is 74.6 Mt. Interestingly, the cultivation area of these cereal crops in 20 years increased by 43.9% in Lithuania, while in Northern Europe, it is only 4.4%.

## 3. Decomposition Dynamics of Cereal Crop Residues

The decomposition of plant litter is widely recognized as a significant contributor of nutrients in both terrestrial and aquatic environments. Furthermore, it is a complex process involving the successive microbial-mediated mineralization and transformation of organic matter. This, in turn, ultimately leads to the release of carbon and nutrients into the biological cycles of the ecosystem [21,22,23]. The temporal changes in plant residue composition observed can be ascribed to the considerable impacts imposed by the following main processes: (a) leaching involves the removal of soluble substances to a lower soil profile, where they undergo subsequent processing by decomposer organisms; (b) fragmentation entails the generation of new surface areas accessible to decomposer organisms through a physical breakdown of large pieces of plant residues into smaller fragments; (c) chemical alteration denotes the chemical modification of the residue, transpiring as decomposer organisms that identify the molecules or selectively utilize constituent portions of it during the production of decomposer biomass [24,25].

**Figure 1:**
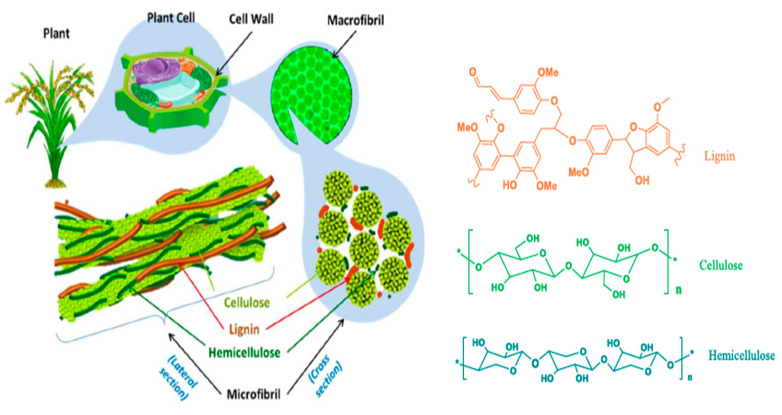
Composition and structure of lignocellulose in plants [27].

The elements of the plant cell wall can be categorized into three main groups based on their resistance to degradation: (i) water-soluble molecules; (ii) cellulose, hemicellulose, and pectin; (iii) lignin and other aromatic compounds (Figure 1) [26]. The primary category consists of small molecules, such as amino acids and sugars, which are readily accessible and susceptible to degradation by the rapidly growing associated microbial community [26,27]. Cereal crop residues, such as wheat straw and corn stalks, often include a substantial amount of cellulose and hemicellulose compared to some other crop residues [28]. In terrestrial and aquatic ecosystems, cellulose is the most common biological substance and the primary component of plant biomass. Every year, plants generate around 180 billion tons of cellulose, which is the world’s biggest organic carbon store, and approximately 1.150 billion tons of it derives from cereal crop residues. This biopolymer consists of linear β-1,4-linked D-glucose chains. The existence of a hydrogen bond between the oxygen atom and the hydroxyl helps to preserve the linear structure of cellulose in the form of a cellulose chain [29,30].

Hemicellulose is another common heterogeneous polymer present in the cell walls of plants, characterized by its predominant equatorial orientation of β-(1),4 backbones. It has an amorphous form and aids in the support of the cellulose crystals. The main constituents of its backbone are xylans, xyloglucans, and mannans, characterized by the presence of branching monomers and short oligomers [31,32]. In comparison to cellulose and hemicelluloses, lignin is a highly complex heteropolymer composed of phenylpropane chains that are linked together by carbon–carbon (C–C) and aryl-ether (C–O–C) links. The structure and content of lignin differ between crops due to its complicated nature, as it lacks a standardized macromolecular structure [33,34]. Table 3 shows that the composition of cereal crop residue typically includes cellulose (26–44%), hemicelluloses (24–36%), and lignin (4–19%). The relative proportions of these compounds significantly differ from crop to crop and even among varieties of the same cereal crop.

In the course of crop decomposition, alterations occur in the chemical composition of the residue as a result of the breakdown of both structural and soluble compounds [44,45,46]. The process is heavily influenced by soil fauna and microorganisms, by which the soluble nutrients could be primarily leached and then either mineralized or immobilized based on the needs of the decomposer communities [47,48,49]. Following the active decomposition stage, there is an acceleration in microbial activity in order to breakdown the labile compounds such as cellulose, hemicellulose, lignin, etc. Microorganisms metabolize these compounds, and consequently, the release of organic carbon occurs mainly in the form of carbon dioxide (CO_2_), a process known as microbial respiration [47,50,51]. It is essential to note that the concentration of organic carbon in the crop residue decreases noticeably throughout this period. This decline is primarily due to organic carbon in the litter serving as the primary energy source for decomposers [52,53].

Grzyb et al. [54] study described in detail the environmental factors influencing crop residue decomposition. According to the study, the microbial decomposition of crop residue in soil and the provision of nutrients to plants is influenced by various environmental factors. These include the composition of plant residues, temperature, soil moisture, texture, and microbiota. Notably, cereal crop residues have a higher carbon-to-nitrogen ratio (C/N) compared to leguminous crops [55]. The elevated C/N may have a significant effect on soil nutrient dynamics and accordingly on the decomposition rate in soil. Hence, the principal three elements governing cereal crop residue decomposition are the physicochemical environment, litter quality, and the composition of the decomposer community [56]. Thus, the interplay between agricultural management practices and soil abiotic and biotic features substantially affects the decomposition process, alongside the impact of residue type.

## 4. Agricultural Practices Enhance the Development of Microbial Communities Associated with Crop Residue Decomposition

The present emphasis on the composition of soil microbial community structures and their alterations resulting from diverse environmental influences is noteworthy [57,58]. Microbial activity is a valid measure of soil quality due to the integral role of soil microorganisms in organic matter degradation and biogeochemical cycles that influence soil fertility [59,60]. Furthermore, the influence of agricultural practices on the formation of microbial consortia responsible for the decomposition of crop residues is profound [61,62]. These practices have both direct and indirect effects on the soil environment, which, in turn, determines the diversity, abundance, and activity of the microorganisms responsible for the decomposition of crop residues, including those derived from cereals.

Conventional tillage, which includes methods like ploughing and turning the soil, has a significant impact on crop residue decomposition. Conventional tillage through physical incorporation of crop residues into the soil can initially accelerate the decomposition by increasing the contact between soil microorganisms and the residues; however, in the long run, repeated soil disturbance may negatively affect soil microbial diversity and biomass [63]. Previous studies suggest that tillage may enhance soil aeration, increase total porosity, and improve oxygen diffusion rates [64,65,66,67]. These changes provide a favorable setting for the development of aerobic microorganisms, thus accelerating the decomposition of soil organic matter, a vital substrate and energy source for soil microorganisms. In line with this, a study found that after two years, the remaining biomass of 14C-labelled wheat residue was significantly lower under conventional tillage (25.8%) compared to reduced tillage (40.0%), corroborating the hypothesis that conventional tillage accelerates residue decomposition [68]. However, it is important to note a general trend of diminished bacterial diversity associated with conventional tillage [69,70,71]. Interestingly, Duan and colleagues [72] suggest that the increase in alpha diversity observed under conventional tillage might result from the disturbance of soil aggregate stability and the subsequent release of physically protected organic matter, which is then utilized by microbes. Nonetheless, it is crucial to acknowledge that the outcomes of such studies are heavily influenced by factors such as the study site, soil types, pedoclimatic conditions, etc.

Conservation tillage, encompassing practices such as reduced tillage, no-tillage, and strip-till, minimizes soil disturbance, maintains soil structure, and increases soil organic matter content that provides a more stable habitat for a wider range of microorganisms [73,74]. The effect of long-term conventional and conservational tillage on fungal community structures study in Northwest China revealed that fungal taxonomic composition was more balanced under conservation tillage. Notably, the relative abundance of *Basidiomycota*, a major fungal group that participates in crop residue decomposition was high under no-tillage compared to ploughing or chisel ploughing tillage treatments, and *Sordariales* and *Mortierellales* increased by a similar proportion in the bulk soil under conservation tillage treatments [75]. It is more likely that intensive soil disturbance from ploughing and turning disrupts the physical structures within the soil, resulting in breaking fungal networks. This, in turn, may significantly impact the diversity and abundance of soil fungal communities.

The application of crop residue management and conservation tillage practices is crucial in increasing the labile carbon content in agroecosystems, which is primarily utilized by the soil microbial population. The incorporation of labile carbon into the soil initiates a succession of enzymatic processes, primarily mediated by cellulases, which collectively degrade the cellulose present in the labile carbon [76,77,78]. However, only limited studies have been conducted to contrast differences in the taxonomy and functionality of the crop residue decomposition associated microbial communities under conventional and conservation tillage practices [79,80]. The study on metagenomic analysis by Vries et al. [81] hypothesized a higher abundance and diversity of cellulase genes in reduced tillage soil compared to conventional soil due to higher substrate availability under the first approach. However, only a few significant differences were observed: genes related to GH48, encoding an enzyme that plays a crucial role in the breakdown of cellulose, were exclusively found in reduced tillage soil, while conventionally tilled soil had more CBM11 genes that help to enhance the ability of cellulolytic enzymes to effectively bind and degrade cellulose. The findings of this study indicate that the intensity of tillage may only have a limited impact on the structure of microbial communities over a period of 20 years. Furthermore, the study suggests that microbial communities within agricultural soils maintain relative stability despite long-term differences in tillage practices.

The relative abundance of oligotrophic bacteria, such as *Acidobacteria*, *Planctomycetes*, and *Verrucomicrobia* tends to be high under conservation agriculture. On the other hand, a conventional practice that mixes the top layers of soil promotes the proliferation of bacterial groups that prefer nutrient-rich environments, like *Actinobacteria* [82]. The cause of this phenomenon is attributed to the increased availability of organic materials to soil microorganisms in conventional farming. In another study tillage and crop residue management emerged as a key determinant of bacterial communities at the genus level responsible for decomposing of wheat residue introduced into the soil. In particular, the relative abundance of genera such as *Promicromonospora*, *Bacillus*, *Sinorhizobium*, and *Lysobacter* increased significantly [83].

Extracellular enzymes operate as direct facilitators of organic matter breakdown, and measuring their activity may accurately reflect microbial nutritional needs [84]. In particular, β-glucosidase (EC 3.2.1.21) is positioned as the final enzyme in cellulase enzymes’ succession, and vital in glucose production [85]. Researchers have used the enzyme’s potential activity as an indicator of organic matter decomposition since β-glucosidase activity responds to the application of organic amendments to soil [86,87,88,89]. Recent research demonstrated that reducing tillage and retaining wheat stubble mulch increased bulk soil β-glucosidase activity by 58% compared to conventional tillage with residue removal over 11 years [90].

## 5. Potential Utilization of Lignocellulolytic Microorganisms in Cereal Crop Residue Decomposition

Microorganisms employ a range of mechanisms, such as the utilization of different enzymes that work in combination, to initiate oxidative attacks on plant litter. Consequently, this serves to reduce the recalcitrance of the lignocellulosic material, hence facilitating subsequent action by depolymerizing enzymes [91]. In recent years, both fungi and bacteria have received increased attention for their ability to secrete a diverse range of lignocellulolytic enzymes.

### 5.1. Lignocellulolytic Activity of Fungi

Fungi are well known for their pivotal role in the soil microbiota, particularly in relation to the process of plant residue breakdown in the soil. Being filamentous by nature, fungi have an advantage in the breakdown of lignocellulosic material since they can create spores fast and prolifically and are assisted by a wide range of enzymes that have complementary catalytic activities [92]. The extracellular enzymatic system consists of hydrolytic enzymes that are involved in the breakdown of polysaccharides, as well as oxidative enzymes that are responsible for causing the deterioration of lignin and the opening of phenyl rings. Within the realm of lignocellulose breakdown, there are three particular groups of fungi, namely soft-rot, brown-rot, and white-rot fungi, each of which demonstrates diverse impacts and degradation techniques [93,94]. Likewise, it was reported that the decomposition process of plant residue in natural ecosystems is greatly aided by the presence of the *Basidiomycota* group [95].

White-rot fungi utilize a diverse range of carbohydrate-active enzymes (CAZymes) that specifically target cellulose, hemicellulose, and pectin [96,97]. Furthermore, they use lignin-modifying enzymes belonging to the AA2 family and include class-II heme peroxidases including lignin peroxidases, versatile peroxidases, and manganese peroxidases [98,99]. These enzymes, along with auxiliary CAZyme oxidoreductases, catalyze oxidative reactions that effectively degrade the complex lignin polymers in plant residue [98,100]. White-rot fungi, belonging to the *Basidiomycota* phylum, have a particular ability to metabolize lignin as their primary source of energy [101]. Furthermore, there has been significant research conducted on the examination of lignocellulosic pretreatment methods, specifically focusing on two distinct classifications: selective and non-selective delignifiers of fungal species of the *Basidiomycota* phylum. The primary focus of selective delignifiers is to specifically target heteropolymeric lignin while scarcely affecting cellulose and hemicellulose components [101,102,103]. The aforementioned attribute renders them more appealing for scientific investigation, as they demonstrate a greater output of lignin-free cellulosic biomass in comparison to non-selective delignifiers. These are mostly responsible for the simultaneous degradation of lignocellulosic biomass structural components [104,105,106]. Several species of white-rot fungi have notable potential in the breakdown of lignin. *Phanerochaete chrysosporium* [100,107] and *Trametes versicolor* [108,109] have been the subject of much research due to their significant biological pretreatment capabilities. These organisms are commonly used as model organisms to gain insights into the process of lignin breakdown. Moreover, *Ganoderma lucidum* [110,111] and *Phlebia* spp. [112,113,114] have also been acknowledged for their ability to produce lignin-degrading enzymes, which enhances their potential to be viable candidates for diverse applications in the area. Xu and colleagues [115] obtained promising results, investigating the efficacy of white-root fungus *Inonotus obliquus* pretreatment for the first time in producing lignocellulolytic enzymes induced by wheat straw, rice straw, and maize stover biomass. The fungus process resulted in the highest lignin loss of 72%, 39%, and 47% within 12 days for wheat straw, rice straw, and corn stover, respectively.

Another essential group of fungi in the degradation of plant residue are brown-rot fungi, which are involved in the breakdown of crop residue. Similarly to forest ecosystems, brown-rot fungi adopt a selective decay strategy that primarily targets the degradation of cellulose and hemicellulose, while mostly modifying lignin rather than completely degrading it [116,117]. In comparison with white-rot fungi, brown-rot fungi do not possess genes for class-II peroxidases. As a result, the breakdown of polysaccharides of plant cell walls mostly occurs via non-enzymatic Fenton reactions, which are produced outside the fungal hyphae [91,118]. In the Hermosilla and colleague study, wheat straw pretreatment by the brown-rot fungus *Gloeophyllum trabeum* resulted in 11.3% weight loss after 40 days, and increased glucose recovery [119]. Another comparative investigation of lignocellulosic biomass degradation revealed that brown-rot fungi *Fomitopsis pinicola* exhibited the highest level of maize stalk mass reduction by 38% at 16 weeks among the species examined. Importantly, the observed effect was preceded by a substantial lag phase, a characteristic that was conspicuously lacking in the degradation patterns reported concerning other species [120]. It is worth noticing that the research on the ability of brown-rot fungi to degrade lignocellulosic biomass, particularly those derived from agricultural ecosystems, is relatively limited.

The significant contributions of lignocellulolytic fungi, particularly white-rot and brown-rot fungi, in the context of sustainable crop residue management, are of utmost importance and should not be underestimated. Initially, they utilize a diverse array of enzymes, efficiently breaking down complex lignocellulosic structures. Moreover, these fungi employ distinct mechanisms, such as selective degradation and the Fenton reaction, to enhance and accelerate the process. The comprehensive nature of this method highlights the essential contribution of these organisms in the natural processes of carbon and nitrogen recycling, hence playing a crucial role in the preservation of ecological equilibrium. In assessing the utilization of fungi for the decomposition of crop residue, it is important to take into account several factors. First and foremost, species specificity is of utmost importance, since different fungi exhibit varying levels of effectiveness and preference for different types of residues. Furthermore, it is essential to verify that the selected fungi do not cause diseases in crops and have positive effects on soil health. Therefore, it is crucial to carry out experimental studies or trials in order to evaluate the efficacy and safety of these fungi in certain agroecosystems, thereby guaranteeing an informed and appropriate strategy in terms of sustainable crop residue management.

### 5.2. Lignocellulolytic Activity of Bacteria

In addition to fungi, the utilization of bacteria for possible biodegradation processes is beginning to gain recognition due to their extensive functional diversity and versatility [121]. That can be explained by the ability to exhibit rapid growth rates and a remarkable tolerance range in terms of temperature, pH, and salinity, enabling them to adapt to a diverse array of environmental conditions [122,123,124,125]. In addition, some bacteria have the ability to meet their nitrogen needs through the process of biological nitrogen fixation [126]. Moreover, bacterial lignocellulases can produce multi enzymatic complexes, which are more adapted for the elaborate breakdown of biomass [127]. Some of the observations show that bacteria increase abundance in the latter stages of the lignocellulose breakdown process, which can be determined by the predominance of complex and recalcitrant carbon sources [128,129]. However, Arcand and colleagues [130] observed temporal variations in the relative abundance of Gram-positive bacteria depending on treatment, in contrast to a consistent decline in Gram-negative bacteria across different treatments. Concurrently, there was a notable increase in the relative abundance of Actinobacteria over time, a trend that persisted across all treatment conditions. Therefore, it is essential to acknowledge one more time that the abundance of bacteria in soil, specifically during the process of plant residue breakdown, is influenced by a complex combination of biotic and abiotic factors. Including nutrient availability, prevailing environmental conditions, inter-species competition, and synergistic associations, as well as the particular types of crops and their residues. The complex relationship described highlights the subtle features that define soil microbial ecology in agricultural ecosystems.

Bacteria possess distinct species and decomposition mechanisms that are adapted for either aerobic [131] or anaerobic conditions in plant lignocellulose breakdown [132]. During the lignocellulose degradation process, aerobic bacteria commence the breakdown by secreting lignocellulolytic enzymes that are capable of targeting biomass [133]. The bacteria initially engage in the hydrolysis of cellulose, resulting in the production of cellobiose, which is then followed by a stage of fermentation [134]. During this phase, the cellobiose molecule undergoes further hydrolysis reactions, leading to the formation of carbon dioxide, hydrogen, and other organic acids [135]. Within the bacterial community, some aerobes, such as *Cellulomonas*, *Bacillus*, *Pseudomonas*, and *Streptomyces*, etc., are recognized as key players in cellulose degradation, contributing significantly to the process of residue decomposition [136,137,138,139].

Aerobic bacteria have numerous significant benefits over anaerobic bacteria in the context of crop residue breakdown in agricultural settings [140,141]. Firstly, aerobic bacteria exhibit a faster rate of breakdown, which can be attributed to the more effective pathways for energy release in the presence of oxygen. Their high efficiency allows them to rapidly break down complex compounds such as cellulose and lignin [142]. This phenomenon occurs as a result of the wide range of enzymes generated by aerobic bacteria, which efficiently break down recalcitrant plant compounds and facilitate complete mineralization. Finally, the aerobic process improves the availability of nutrients in forms that are easier for plants to absorb. For instance, nitrogen is released in the form of nitrate, which is easily assimilated by plants, in contrast to the ammonium released by anaerobic processes [143,144,145].

In the anaerobic degradation of lignocellulose by bacteria, sugars are transformed into alcohol or acids, leading to the generation of biogas through subsequent anaerobic digestion [146]. This process involves various microorganisms, notably methanogens and acetogens, which are capable of utilizing cellulose. While CO_2_ is the primary byproduct of microbial cellulose degradation, methane (CH_4_) is also produced under anaerobic conditions [134]. The genus *Clostridium* is well recognized as a highly researched group for anaerobic degradation of lignocellulose, mostly due to its exceptional ability to efficiently breakdown cellulose [147,148]. This particular genus possesses the ability to produce complex enzymes known as cellulosomes, which exhibit a high level of efficiency in the process of breaking down cellulose and hemicellulose [146,149]. Moreover, anaerobic bacteria break down cellulose utilizing complex cellulase systems such polycellulosomes, while aerobic bacteria use a synergistic free cellulase system to utilize cellulose as a carbon and energy source by secreting different types of endo- and exo-acting enzymes.

### 5.3. Lignocellulolytic Activity of Actinobacteria

Another group of microorganisms that display characteristics that bear resemblance to both bacteria and fungi are actinobacteria. Nevertheless, the resemblance between actinomycetes and fungi is only superficial, and they possess sufficient distinctive characteristics to definitively classify them within the bacterial kingdom [150]. The majority of filamentous actinomycetes that frequently occur belong to the *Streptomyces* and *Micromonospora* families. Usually, actinomycetes are known for their ability to break down complex carbon and nitrogen compounds [151,152]. In soil, organic residues are initially decomposed by bacteria and fungi with actinomycetes subsequently taking over due to their comparatively slower growth and activity rates. Moreover, they have a crucial function in the subsequent decomposition of humus in the soil [153,154]. Actinobacteria communities exhibit a wide range of hydrolytic enzymes, such as β-glucosidase, cellobiohydrolase, ligninase, acetyl xylan esterase, and arabinofuranosidase. These enzymes, along with their associated supramolecular cellulosomes, are crucial for breaking down plant residues [155,156,157].

Additionally, the high C/N ratio in cereal crop residues constrains the nitrogen availability for microbial reproduction. However, the nitrogen-fixing capability of actinobacteria potentially enhances nitrogen availability during cereal residue decomposition driven by microbes [158,159,160]. Notably, this group of microorganisms have the ability to inhibit the growth of other species by producing antibiotics [161]. These ecological and physiological attributes collectively indicate a wide adaptation of actinobacteria communities in crop residue decomposition and soil carbon sequestration. 

The key genera engaged in the process of degrading lignocellulose biomass include *Streptomyces*, *Micromonospora*, *Thermobifida*, *Thermomonospora*, *Actinomadura*, *Nocardia*, and others [18,162,163,164,165,166,167,168]. A study on wheat straw biodegradation by *Streptomyces viridosporus* T7A, revealed lignin and hemicellulose removal, carbonyl and methoxyl group modifications, and a significant guaiacyl unit reduction [169]. Another research conducted by Gong and his colleagues on the characterization of maize-straw-degrading actinomycetes revealed that a consortium composed of the three *Streptomyces* spp. showed a decomposition rate of 51.60% after 77 days, significantly reducing the content of recalcitrant components in the maize straw [170]. A metatranscriptomic analysis of compost-derived microbial communities enriched on rice straw under thermophilic and mesophilic conditions showed significant overexpression of enzymes from glycoside hydrolase family 48 and carbohydrate-binding modules families 2 and 33 in the thermophilic community, predominantly expressed by the actinobacteria genus *Micromonospora* [171].

Another study investigated the process of breaking down lignocellulose and specifically focused on the lignin-degrading ability of peroxidase Tfu-1649 secreted by *Thermobifida fusca* BCRC 19214, particularly in synergy with xylanase Tfu-11 substantially enhanced the degradation of lignocellulosic biomass [166].

The efficacy of cereal crop residue breakdown by various microbial inoculants varies depending on parameters such as application rate, timing of inoculation, type of cereal crop residue, etc. The efficiency of the microorganisms was assessed by measuring the percentage of mass loss of residues. The results are summarized in Table 4. The secretion of hydrolytic enzymes positions actinomycetes as a principal group among soil microorganisms that are responsible for organic matter decomposition. As decomposers, they are adept at breaking down resilient lignocellulose from crop residues including the most recalcitrant structures such as lignin. Furthermore, the nitrogen-fixing capability of certain species enhances the decomposition of cereal crop residues, which typically have low nitrogen content.

## 6. Research Gaps and Future Directions

Further investigation is required to thoroughly examine the influence of different cereal crop residue impacts under different agricultural practices on soil microbial community structure and functionality responsible for the decomposition processes. Through the utilization of metagenomic and metatranscriptomic approaches, it is possible to discover the presence and functionality of microorganisms involved in the decomposition processes of crop residues in the soil. While short-term studies provide significant insights, there is a lack of knowledge regarding the long-term effects of different agricultural practices on microbial communities and residue decomposition in different pedoclimatic conditions. Moreover, an increase in the number of long-term experiments would facilitate the performing of a high-quality meta-analysis, which might provide the overall effect of specific agricultural practices on soil microbial community diversity associated with crop residue decomposition. It is currently unclear how shifting climatic conditions, including rising temperatures and altered precipitation patterns, impact soil microbial communities and the decomposition of residues in various agricultural systems.

Studies should be initiated on developing artificial microbial consortia with a balanced composition of microorganisms that are able to secrete a wide range of enzymes to degrade both simple and complex compounds in crop residues. Furthermore, it is essential to comprehend the effect of microbial inoculants on the diversity and dynamics of native microbial communities in the soil. Research should examine whether inoculants cause shifts in microbial communities and if these shifts have enduring consequences for soil health.

## 7. Materials and Methods

**Figure 2:**
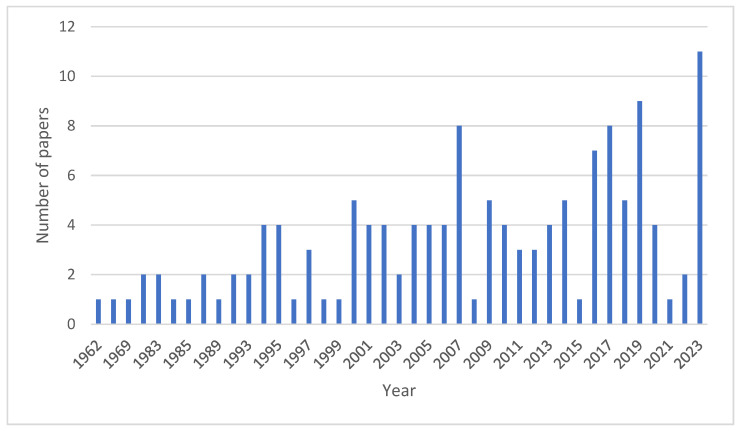
Number of papers on the keywords “microbial crop residue decomposition” published between 1962 and 2023.

**Figure 3:**
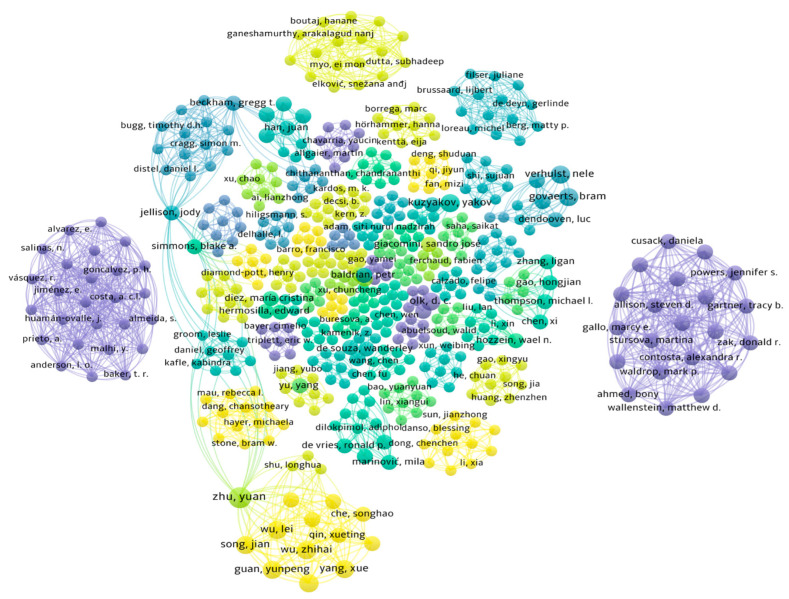
Bibliographic network of the literature used.

The present study gives an indirect estimation of cereal crop residue production in the Northern European region and Lithuania based on grain production. Cereal crop residue (CCR) production is calculated using the equation (CCR = GP × r), where CCR stands for cereal crop residue production, GP stands for grain production adopted from FAOSTAT [19] official website, and r stands for straw/grain ratio of crops adopted from the literature [183]. In this review, the methodology used was according to Toronto and Remington [184], which consists of the following stages: (1) problem formulation stage, to justify the study stated; (2) literature search stage, in which data are collected using a comprehensive and replicable search strategy; (3) data evaluation stage, in which the methodological quality and relevance of the selected literature are appraised; (4) data analysis stage, which includes data abstraction, comparison, and synthesis; and (5) presentation stage, in which the interpretation of findings and implications for research. Web of Science core collection, Bielefeld Academic Search engine (BASE), OpenAlex catalog, Scopus, MDPI, and ScienceDirect were searched for relevant scientific literature. The following keywords were used to search for relevant literature: crop residue, cereal crop residue, microbial crop residue decomposition, cellulose, hemicelluloses, lignin, soil, crop residue management, tillage practices, β-glucosidase activity, and lignocellulose-degrading microorganisms. The search of the global research system OpenAlex catalog (https://openalex.org, accessed on 25 January 2024) [185] using the keyword “microbial crop residue decomposition” with filters “crop residue” and “decomposition” yielded 138 papers published between 1962 and 2023 (Figure 2). The total number of the literature sources used in this review is 186. The bibliographic network of all literature sources used in this review is shown in Figure 3, which was created to summarize and illustrate the connection pattern using VOSviewer version 1.6.20 [186].

## 8. Conclusions

This review comprehensively addresses the primary impacts of agricultural practices on soil microbial community involved in decomposing cereal crop residues. The evidence presented highlights the significant influence of agricultural practices and residue types, particularly in shaping the microbial decomposer community. Notably, conventional tillage practices can initially accelerate the decomposition rate by breaking down soil aggregates and incorporating crop residues into the soil which increases their contact with soil microorganisms. However, the disruption of soil structure can adversely affect microbial habitats, thus causing a shift towards bacterial dominance in soil due to the disturbance of the fungal network. Therefore, the conservation agricultural practices that support increased fungal diversity and a more balanced bacteria-to-fungi ratio, which is often associated with healthier soil systems have a high probability of developing efficient soil microbial consortia responsible for crop residue decomposition in soil. Additionally, we have systematically elucidated the mechanisms of lignocellulolytic microorganisms and their enzymatic activities in accelerating the decomposition of recalcitrant cereal crop residues. The selection of crop residue type-specific microbial consortia able to effectively decompose under varied pedoclimatic and environmental conditions is crucial.

## Figures and Tables

**Table 1:** Grain and cereal crop residue production in 2001, 2011, and 2021 in Northern Europe (area and grain production are calculated from FAO).

Cereal Crops	Area (10^3^ Kha)			Production (10^3^ Kt)			Residue Production (10^3^ Kt)		
	2001	2011	2021	2001	2011	2021	2001	2011	2021
Barley	3892.5	3123.3	3100	17,213	14,865.3	15,764.350	25,820.1	22,298	23,646.5
Maize	4.7	20.2	26	10.9	127.2	164.850	10.9	127.2	164.9
Oats	1126.2	881.8	1065.6	3859.3	3284.9	3739.330	3859.2	3284.9	3739.3
Rye	324.5	203.5	279.6	985	733	1361.719	1477.5	1099.4	2042.6
Wheat	3539.9	4543.2	4806.6	21,793.7	27,699	30,037.550	32,690.6	41,548.5	45,056.3
Total	8887.9	8772	9277.9	43,862.3	46,709.3	51,067.799	63,858.4	68,357.9	74,649.6

**Table 2:** Grain and cereal crop residue production in 2001, 2011, and 2021 in Lithuania (area and grain production are calculated from FAO).

Cereal Crops	Area (10^3^ Kha)			Production (10^3^ Kt)			Residue Production (10^3^ Kt)		
	2001	2011	2021	2001	2011	2021	2001	2011	2021
Barley	336	252.7	144.7	776.2	759.8	500.4	1164.3	1139.7	750.6
Maize	4.7	9.6	17.9	10.9	71.9	104.7	10.9	71.9	104.7
Oats	47.9	63.2	92.4	84.3	128.5	170.3	84.3	128.5	170.3
Rye	111.3	42	26.1	231.1	85	63.5	346.7	127.5	95.2
Wheat	352.2	551.1	944.2	1076.3	1869.3	4248.9	1614.5	2804	6373.3
Total	851.6	918.6	1225.3	2178.8	2914.5	5087.7	3220.6	4271.6	7494

**Table 3:** Chemical components of the world’s most abundant cereal crop residues. Composition is represented as % weight based on dry matter.

Crop Residue	Cellulose (%)	Hemicellulose (%)	Lignin (%)	Reference
Wheat straw	34–39	26–32	16–19	[35,36]
Oat straw	27–34	24–33	9–15	[37,38,39]
Rye straw	26–36	28–34	13–21	[40,41]
Maize stalks	38–43	26–31	4–10	[36,42]
Barley straw	35–44	27–36	14–18	[41,43]

**Table 4:** Summary of microbial inoculant efficiency for degradation of cereal crop residues.

Microorganism	Residue Type	Method	Days	Mass Loss, %	Enzyme(s) Evaluated	Reference
*Trichoderma reesei*	Rice straw, bran	Solid-state fermentation	10	51.16	Laccase, xylanase, β-Glucosidase, cellobiohydrolase, endoglucanase	[172]
*Trichoderma harzianum*	Rice straw	In situ	28	23.69	-	[173]
*Aspergillus niger*	Rice and wheat straw (4:1)	Solid-state fermentation	10	16	CMCase, endoglucanase, cellobiase, β-1,4-xylanase	[174]
*Phanerochaete chrysosporium*	Maize stover	Solid-state fermentation	28	21	-	[175]
*Ganoderma lobatum*	Wheat straw	Solid-state fermentation	40	21.04	β-glucosidase	[176]
*Cellulomonas* sp.	Rice straw	Submerged fermentation	4	49.3	β-glucosidase, endoglucanase, exoglucanase, xylanase, lignin peroxidase, manganese peroxidase, laccase.	[177]
*Bacillus* sp.	Wheat bran	Submerged fermentation	7	60	Cellulase, endoglucanase, xylanase, laccase, mannase	[178]
*Streptomyces* sp.	Barley straw	Submerged fermentation	7	60.55	Exoglucanase, endoglucanase, β-glucosidase	[179]
*Enterobacter* sp.	Rice straw	Submerged fermentation	7	45.52	Endoglucanases, exoglucanase, xylanase	[180]
*Ganoderma lobatum* + *Gloeophyllum trabeum*	Wheat straw	Solid-state fermentation	20	15.52	β-glucosidase	[176]
*Cellulomonas* ZJW-6 + *Acinetobacter* DA-25	Rice straw	Submerged fermentation	4	57.62	β-glucosidase, endoglucanase, xylanase, lignin peroxidase, laccase, manganese peroxidase, β-glucosidase	[181]
*Streptomyces* sp. G1T + *Streptomyces* sp. G2T + *Streptomyces* sp. G3T	Maize stalk	solid-state fermentation	119	66.37	-	[170]
*Citrobacter freundii* so4 + *Sphingobacterium multivorum* w15 + *Coniochaeta* sp. 2T2.1	Wheat straw	Submerged fermentation	10	12.82	-	[182]

## Data Availability

Not applicable.
